# Three-dimensional printed bioresorbable scaffold for maxillofacial bone reconstruction: A Scoping Review

**DOI:** 10.1590/0103-6440202406112

**Published:** 2024-10-25

**Authors:** Carolina Mendonça de Almeida Malzoni, Jovânia Alves Oliveira, Lélio Fernando Fereira Soares, Marcella Cunha Chimirri, Daniel Augusto de Faria Almeida, Suzane Cristina Pigossi, Elcio Marcantonio

**Affiliations:** 1 Department of Diagnosis and Surgery, School of Dentistry at Araraquara, UNESP - São Paulo State University(FOAr/UNESP), Araraquara, São Paulo, Brazil.; 2 Department of Periodontology and Implantodontology, School of Dentistry, Federal University of Uberlândia - UFU, School of Dentistry, Uberlândia, MG, Brazil.; 3 School of Dentistry, Alfenas Federal University (Unifal-MG), Alfenas, Minas Gerais, Brazil.

**Keywords:** Three-Dimensional Printing, Bone graft, Bone regeneration, Biocompatible materials

## Abstract

This scoping review aimed to provide an overview of current advancements in virtual planning and custom-made 3D-printed bioresorbable scaffolds, and to evaluate their clinical outcomes in maxillofacial reconstructive surgeries. Electronic searches of PubMed, EMBASE, Web of Science, Scopus, and Cochrane Library databases were conducted for publications up to June 2024. Included in the review were reports evaluating patients who underwent maxillofacial bone defect reconstruction using virtual planning and custom-made 3D-printed bioresorbable scaffolds. Data on postoperative complications, new bone formation, scaffold resorption, dental implant success/survival, and patient satisfaction were collected. The electronic search found 5799 results (3438 unique citations). A total of 54 studies were evaluated for full-text reading, of which 41 were excluded based on the inclusion criteria. Thirteen studies (6 case reports, 5 case series, one prospective clinical study and one randomized clinical trial) were included. These studies assessed the effectiveness of 3D-printed scaffolds in reconstructing maxillofacial defects, bone augmentation for dental implant placement, and regeneration of periosseous defects. Most of the 3D-printed scaffolds were biocompatible and did not cause local or systemic adverse events. However, some postoperative complications were reported, including graft exposure, wound dehiscence, and local infection. Overall, the 3D-printed scaffolds demonstrated favorable dimensional compatibility with deformities, provided durable support, promoted bone formation, achieved adequate bone union with host bone tissues, and supported dental implant placement without additional guided bone regeneration. In conclusion, custom-made 3D-printed bioresorbable scaffolds, guided by virtual planning, present a promising option for maxillofacial reconstruction due to their accuracy, osteoconductivity, and biocompatible properties.

## Resumo

Esta revisão de escopo teve como objetivo fornecer uma visão geral dos avanços atuais no planejamento virtual e no uso de enxertos bioabsorvíveis personalizados impressos em 3D, e avaliar seus resultados clínicos em cirurgias reconstrutivas maxilofaciais. Foram realizadas buscas eletrônicas nas bases de dados PubMed, EMBASE, Web of Science, Scopus e Cochrane Library para publicações até junho de 2024. Foram incluídos na revisão estudos que avaliaram pacientes submetidos à reconstrução de defeitos ósseos maxilofaciais usando planejamento virtual e enxertos bioabsorvíveis personalizados impressos em 3D. Foram coletados dados sobre complicações pós-operatórias, nova formação óssea, reabsorção do enxerto, sucesso/sobrevivência do implante dentário e satisfação do paciente. A busca eletrônica encontrou 5799 resultados (3438 citações únicas). Um total de 54 estudos foram incluídos para leitura completa do texto, dos quais 41 foram excluídos com base nos critérios de inclusão. Treze estudos (6 relatos de casos, 5 séries de casos, 1 estudo clínico prospectivo e 1 estudo clínico controlado randomizado) foram incluídos. Esses estudos avaliaram a eficácia de enxertos impressos em 3D na reconstrução de defeitos maxilo-faciais, aumento ósseo para colocação de implantes dentários e regeneração de defeitos ósseos periodontais. A maioria dos enxertos impressos em 3D foram considerados biocompatíveis e não causaram eventos adversos locais ou sistêmicos. Entretanto, algumas complicações pós-operatórias foram relatadas, incluindo exposição do enxerto, deiscência da ferida e infecção local. No geral, os andaimes impressos em 3D demonstraram compatibilidade dimensional favorável com deformidades, forneceram suporte durável, promoveram a formação óssea, alcançaram uma união óssea adequada com os tecidos ósseos hospedeiros e permitiram a instalação de implantes dentários sem a necessidade de regeneração óssea guiada adicional. Esta revisão pode concluir que os enxertos bioabsorvíveis personalizados impressos em 3D, guiados por planejamento virtual, apresentam uma opção promissora para reconstrução maxilo-facial devido à sua precisão, osteocondutividade e propriedades biocompatíveis.

**Figure:**
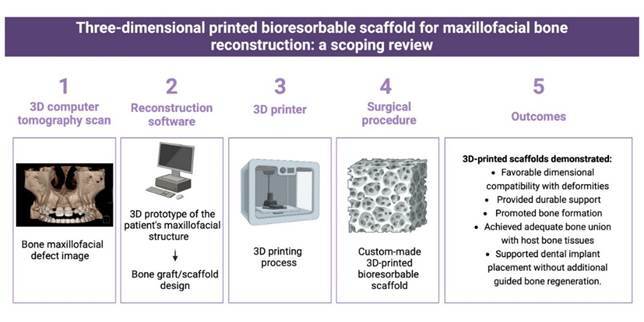

## Introduction

Reconstructive surgeries for maxillofacial defects due to trauma or resulting from ablative procedures are crucial in restoring anatomic structures, appearance, and tissue functions^1^. Similarly, in many cases, alveolar ridge deficiency resulting from bone resorption after tooth extraction requires primary augmentation before dental implant placement^2^. Until now, autogenous bone grafts remain the ‘‘gold standard’’ for these procedures, providing their osteogenic, osteoinductive, and osteoconductive properties^3^. However, the need for a second surgical site to harvest the grafts from an unaffected area drastically increases the procedure morbidity, including postoperative pain, infection risk, ambulatory difficulty, and sensory abnormalities. Furthermore, high graft resorption rate, limited availability, and anatomical limitations are deemed significant limitations^2^. The need to manually sculpt the bone blocks into the complex tridimensional (3D) defect configuration is also highlighted as a significant disadvantage of autogenous bone grafts in these procedures, increasing its complexity and surgery time^4^.

Allografts and synthetic bones have been used to treat craniofacial deformities as substitutes for autogenous bone grafts^5^ ^,^ ^6^ ^,^ ^7^. Nonetheless, concerns over biological contamination and ethical considerations related to the trade of bodily tissues have restricted the use of allografts^5^. Conversely, synthetic bones offer advantages over autografts and allografts regarding safety and invasiveness since they eliminate contamination risks and the need for bone harvesting. However, similarly to autogenous bone, both types of grafts require manual shaping during surgery, leading to diminished precision and suboptimal aesthetic results^6^.

**Figure 1:**
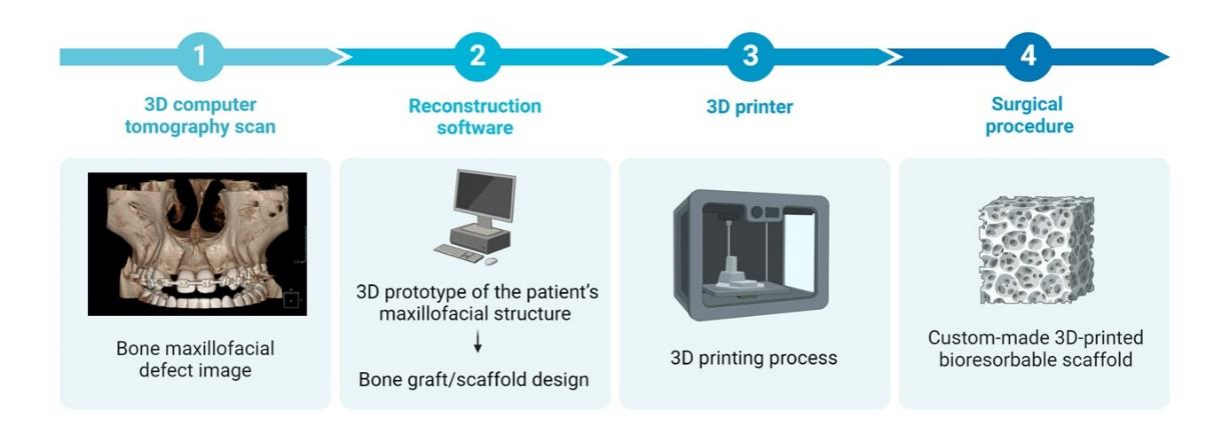
Three-dimensional printed bioresorbable scaffold obtention sequence.

Recently, virtual planning and 3D fabrication of custom prototypes, surgical guides, templates, implants, grafts, and scaffolds have become tools of interest in cranio-maxillofacial surgeries to overcome these limitations^7^. Using a 3D computed tomography (CT) scan, it's possible to create a 3D prototype of the patient’s maxillofacial structure by transferring the files to specific reconstruction software. Advanced computer-aided design (CAD) software can design a custom-made bone graft based on this 3D model, ensuring it fits precisely into the intended site (Figure 1). This approach guarantees a more effective, patient-specific treatment with a simplified surgical procedure that consumes less time^8^.

Three common 3D fabrication methods include cutting, casting, and layer manufacturing^6^. In particular, layer manufacturing (such as fused deposition modeling, selective laser sintering, stereolithography, and inkjet printing) involves the fabrication of objects through material deposition, allowing the construction of objects layer-by-layer from a digital CAD file^8^. Unlike the other two methods, layer manufacturing techniques can be mimicked. Unlike the other two methods, layer manufacturing techniques can mimic complex external shapes and internal structures to be reconstructed from maxillofacial deformities. Moreover, the possibility of manufacturing internal interconnective pores and vessels in personalized scaffolds, medical implants, and grafts allows the possibility of vascularization and cell invasion of these synthetic biomaterials^9^.

Layer manufacturing technology holds the potential to offer reduced surgical time, morbidity, and highly customized grafts for reconstructing maxillofacial defects in a convenient, rapid, and cost-effective manner^10^. Various materials and processes have been investigated and documented for this purpose. Nonbiodegradable scaffolds have been reported, made of titanium or polyetherketoneketone, using 3D fabrication technology^11^. Nonetheless, the implanted nonbiodegradable scaffolds are enduring and could potentially induce inflammation and infection at the implantation site^10^. In response to these constraints, biodegradable or bioabsorbable materials have garnered attention for their ability to possess rigidity and biocompatibility, promote bone regeneration, and reduce the risk of foreign body reactions^12^. However, concerns about material resistance, vascularization, resorption, fixation methods, indications, and host responses are some of the topics under review.

Thus, this scoping review presents the current ‘state of the art’ about virtual planning and custom-made 3D-printed bioresorbable scaffolds and their reported clinical results applied to maxillofacial reconstructive surgeries.

## Materials and methods

### Protocol and registration

This study was registered with the International Platform of Registered Systematic Review and Meta-analysis Protocols (INPLASY) (registration number: INPLASY202460096) and adhered to the Preferred Reporting Items for Systematic Reviews and Meta-Analyses for Scoping Reviews (PRISMA-ScR) guidelines for reporting^13^.

### Focused question

A specific review question was elaborated by PICO (population; intervention; comparator; outcome): "Does the utilization of 3D-printed bioresorbable scaffolds effectively promote bone regeneration in maxillofacial bone defects?"

### Eligibility criteria

The following inclusion criteria were adopted based on the following PICO criteria: (P) Population: patients with maxillofacial bone defects; (I) Intervention: maxillofacial bone defects reconstruction using virtual planning and custom-made 3D-printed bioresorbable scaffolds; (C) Comparator: bone defects reconstruction using different types of bone grafts; (O) Outcomes: postoperative complications, new bone formation, scaffold resorption, dental implant success/survival and patient satisfaction; (S) Study design: clinical studies [including randomized clinical trials (RCTs), controlled clinical studies, cohort studies (prospective or retrospective), case series and case reports] reporting data about using 3D-printed bioresorbable scaffolds for the reconstruction of maxillofacial bone defects. Moreover, only articles in the English language were included.

Original research articles that did not follow the above criteria were excluded from this scoping review. Moreover, letters to the editor, conference proceedings, protocol articles, historical reviews, preclinical studies, and unpublished articles were also excluded.

### Information source and search

Electronic searches of PubMed, EMBASE, Web of Science, Scopus, and Cochrane Library databases were conducted for publications up to June 2024. The search strategies were formulated using the Medical Subject Headings (MeSH) and Embase Subject Headings (Emtree). Boolean operators (AND and OR) combined the descriptors and improved the search strategy through different combinations, respecting each database syntax rule (Appendix). Grey literature (Google Scholar database) was also searched. No filters were utilized in the search strategy.

### Selection of sources of evidence

Publications found in all electronic databases were transferred to the EndNote Program™ X9 version (Thomson Reuters, New York, NY, USA, 2018) to remove duplicate references. Then the results were exported to Rayyan QCRI software (Qatar Computing Research Institute, Doha, Qatar) for selection by titles and abstracts. The studies were selected by two independent researchers (CMAM and JAO). Titles and abstracts of retrieved articles were screened for eligibility, considering the inclusion/ exclusion criteria described above, and irrelevant studies were excluded. Full texts of the studies that met the eligibility criteria were selected and were accessed by both authors for inclusion. Disagreements between the investigators were resolved by consensus or were referred to a third review author (SCP) for the final decision. Studies that met the selection criteria were processed for data extraction. The articles excluded in the full-text analysis were listed separately, and the reasons for exclusion were specified.

### Data charting and synthesis of results

Two investigators (CMAM and JAO) independently read all studies and extracted the following data from all included studies using a standardized spreadsheet: (a) study design; (b) sample size; (c) patients’ gender and mean age; (d) maxillofacial defect region; (e) alloplastic material type; (f) graft pore size and porosity; (g) graft dimension; (h) software and printer type; (i) fixation type; (j) postoperative complications; (k) Follow-up; (l) Outcomes (bone regeneration, dental implant success/survival and patients satisfaction).

### Synthesis of results

Based on the review objective and question, a logical and descriptive summary of the results was made. A table was developed describing the characteristics of the included studies and the key information relevant to the review question (Appendix).

## Results

### Selection of sources of evidence

**Figure 2:**
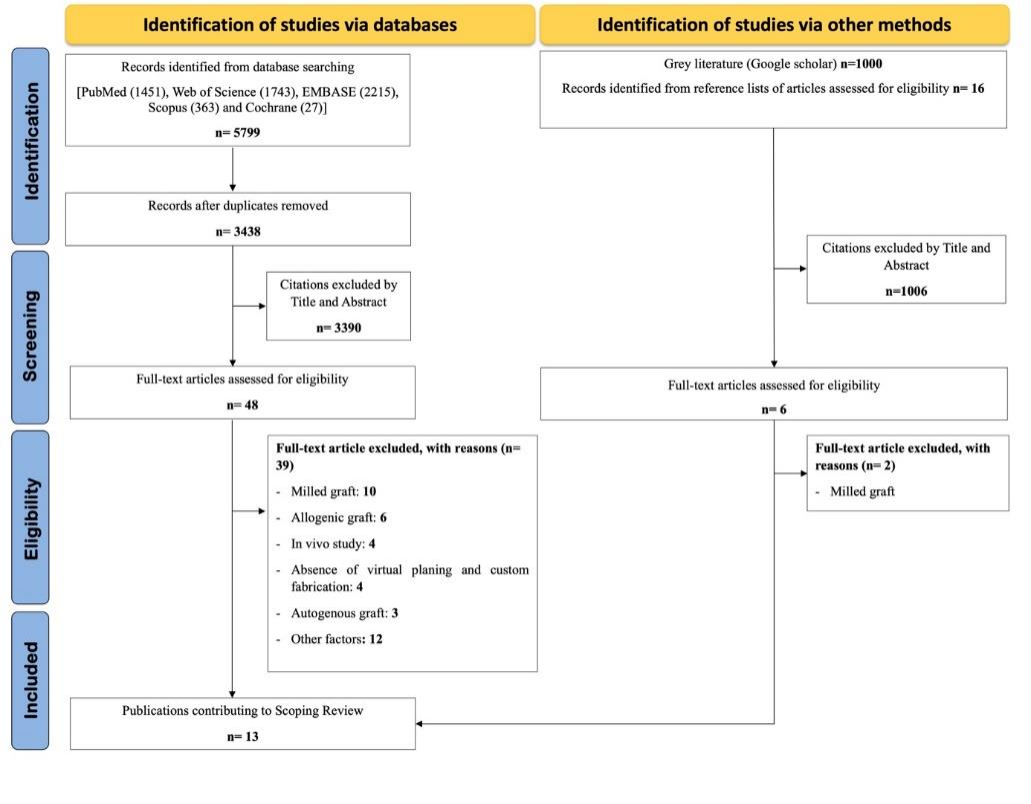
Literature search flowchart.

The electronic database search found 5799 results, with 3438 unique citations. A total of 54 publications (48 publications obtained from the database search and 6 publications obtained through other search methods) were evaluated for full-text reading, of which 41 were subsequently excluded based on the inclusion criteria (Figure 2). The exclusion motivation for each study is shown in Appendix. The remaining 13 studies were included in the scoping review (Appendix).

### Characteristics of sources of evidence

Among the 13 studies included, 6 were case reports, 5 were case series, one was a prospective clinical study, and one was classified as an RCT. A total of 132 patients with maxillofacial defects underwent treatment using custom-made 3D-printed bioresorbable scaffolds. Six studies ^6^ ^,^ ^10^ ^,^ ^12^ ^,^ ^14^ ^,^ ^16^ assessed the effectiveness of the 3D scaffolds for reconstructing maxillofacial defects in various regions, including the maxilla (15 partial or total defects and one alveolar cleft defect), mandible (24 defects), chin (15 defects), zygomatic bone (4 defects), orbital floor (2 defects) and frontal bone (1 defect). Six studies^8^ ^,^ ^17^ ^,^ ^18^ ^,^ ^19^ ^,^ ^20^ ^,^ ^21^ utilized the 3D scaffolds for bone augmentation prior to dental implant placement, while one study^9^ employed the 3D scaffolds for regenerating periosseous defects. The minimum postoperative follow-up duration was 1 month, while the maximum was 7 years.

Regarding the type of alloplastic material used for 3D-printed bioresorbable scaffold production, polycaprolactone (PCL) polymer was the most commonly utilized, without any other alloplastic material^14^ ^,^ ^19^ ^,^ ^22^. PCL was also used in combination with beta-tricalcium phosphate (β-TCP)^12^ or hydroxyapatite (HA)^9^. Other alloplastic materials utilized included α-TCP alone ^6^ ^,^ ^23^, β-TCP^20^, nano-HA^2^, HA^16^, calcium phosphate cement^24^ and HA associated with TCP^8^ ^,^ ^21^. Some studies reported the use of additional biomaterials/biological agents in conjunction with 3D-printed scaffolds to enhance the biological response, including autologous bone^20^, demineralized bone matrix^2^ ^,^ ^12^ calcium phosphate^12^, collagen membrane^2^ ^,^ ^20^, bone marrow mesenchymal stem cells (BMSCs)^22^, platelet-rich fibrin (PRF)^2^, platelet-rich plasma (PRP)^19^, concentrated growth factors (CGFs)^2^, recombinant human platelet-derived growth factor-BB (rhPDGF-BB)^9^ and recombinant human bone morphogenetic protein-2 (rhBMP-2)^19^. Only 7 studies^2^ ^,^ ^8^ ^,^ ^12^ ^,^ ^14^ ^,^ ^21^ ^,^ ^22^ ^,^ ^24^ provided data on the pore size of the 3D-printed scaffolds, with sizes ranging from 300 to 500 μm in 4 studies^8^ ^,^ ^12^ ^,^ ^22^ ^,^ ^24^ and 900 to 2250 μm in 3 studies^2^ ^,^ ^14^ ^,^ ^21^. Additionally, four studies reported the porosity of the 3D-printed scaffolds, with values of 50% in 2 studies^12^ ^,^ ^22^, 60% in 1 study^8^, and 70 to 80% in 2 studies^19^ ^,1)^. The fixation of the 3D-printed scaffolds typically involved the use of plates, screws, pins (constructed from titanium, hydroxyapatite/poly-l-lactide, or poly-D and L-lactic acid), and/or wire steel, as reported in 6 studies^9^ ^,^ ^12^ ^,^ ^16^ ^,^ ^20^ ^,^ ^22^ ^,^ ^24^. Three studies used sutures ^6^ ^,^ ^8^ ^,^ ^23^ while three did not employ any fixation methods^2^ ^,^ ^19^ ^,^ ^21^.

### Synthesis of results

Most 3D-printed scaffolds utilized in the included studies were biocompatible and did not induce local or systemic adverse events. However, some postoperative complications were reported, including graft exposure (9 of 74 sites)^2^ ^,^ ^9^ ^,^ ^16^ ^,^ ^23^, wound dehiscence (1 of 8 sites)^12^, and local infection (4 of 20 sites)^23^. In seven studies, the defects were repaired without any abnormal findings^6^ ^,^ ^8^ ^,^ ^14^ ^,^ ^19^ ^,^ ^20^ ^,^ ^21^ ^,^ ^24^.

Overall, the 3D-printed scaffolds utilized in maxillofacial defect reconstruction^6^ ^,^ ^10^ ^,^ ^12^ ^,^ ^14^ ^,^ ^15^ ^,^ ^16^ exhibited favorable dimensional compatibility with deformities, provided durable support, enhanced bone formation [assessed through computed tomography (CT) images], and achieved adequate bone union between the artificial bones and host bone tissues. Two studies^6^ ^,^ ^15^ reported that the patients were satisfied with the treatment outcomes. In addition, using 3D scaffolds for bone augmentation in the maxilla^8^ ^,^ ^17^ ^,^ ^18^ ^,^ ^21^ and mandible^19^ ^,^ ^20^ defects promoted bone formation (confirmed by histological analysis and CT images). It allowed dental implant placement without additional guided bone regeneration. On the other hand, only one case report^9^ described the treatment of a periosseous defect using 3D-printed scaffolds; after 13 months the site showed a larger dehiscence and wound failure, necessitating entire scaffold removal.

## Discussion

Managing complex maxillary defects with traditional reconstruction approaches is challenging when restoring the original 3D bone structure is necessary^12^. As an alternative to conventional methods, this review showed that the use of custom-made artificial bones using a 3D layered manufacturing (3D printing) process enables the fabrication of scaffolds with customized patient- and site-specific forms, geometries, and porosity using biocompatible and bioresorbable materials. Collectively, findings from the included studies indicate that biodegradable printing materials are now viable for the personalized reconstruction of complex maxillofacial defects, yielding satisfactory outcomes by providing durable support and enhancing bone formation.

One of the most important characteristics of scaffolds is their chemical composition. It is recognized that using non-biodegradable materials heightens the risk of inflammation, infection, and potential implant protrusion^14^. The utilization of biodegradable and clinically safe polymers has been proposed to address these constraints. In this review, PCL, either alone or in conjunction with other biomaterials and/or biological agents, was the most commonly used material in 3D-printed scaffold fabrication. PCL safely degrades into carbon dioxide and water and offers a suitable scaffold for guided bone regeneration due to its favorable mechanical properties and biocompatibility, with a slower degradation rate (2-3 years)^12^. In this review, the utilization of PCL 3D-printed scaffold proved unsuccessful in one case report of periosseous defect regeneration^9^. According to the authors, the slow resorption profile of the PCL scaffold, associated with a bulky design, restricted bone regeneration, leading to dehiscence, exposure, and subsequent microbial contamination around teeth after 12 months.

Incorporating β-TCP into PCL has been demonstrated to enhance the mechanical properties of the scaffold and promote osteogenic cell proliferation, differentiation, and mineralization^25^. A scaffold with a ratio of 80:20 PCL:β-TCP, 50% porosity, and 500 μm pore size was found to be effective in promoting early bone growth and ensuring durability in 8 cases of maxillary defects in this review^12^. TCP is a bioceramic material with chemical properties resembling bone minerals and exhibits excellent osteoconductivity^12^. Additionally, TCP displays an unpredictable biodegradation profile lasting 6 to 24 months. According to Yeo, Rai^26^, the PCL-20% TCP scaffold gradually degraded within 6 months while maintaining pore interconnectivity for the formation of the newly mature bone. This initial degradation releases calcium ions, which enhance mineralization by facilitating the osteogenic differentiation of adipose-derived stem cells. Saijo, Igawa^6^ reported 10 successful cases of maxillofacial reconstruction using 3D-printed scaffolds containing α-TCP, wherein partial union between host bone and graft was observed at 12 months.

The use of synthetic HA in the fabrication of 3D-printed scaffolds was also reported in this review. HA exhibits favorable biocompatibility and osteoconductive characteristics owing to its chemical similarity to alveolar bone. It is the least soluble form among naturally occurring calcium phosphate salts, providing an osteoconductive scaffold with notable resistance to physiological resorption^17^. Following HA implantation, forming a thin apatite layer on the material surface facilitates the integration between the host bone and the material. Mangano, Giuliani^8^ described the fabrication of a 3D-printed scaffold using HA (30%) combined with TCP (70%) for regenerating maxillary buccal plate defects. Assessment via microcomputed tomography revealed a decrease in biomaterial volume of over 23%, with newly formed bone accounting for more than 57% of the overall mineralized tissue after a 7-year follow-up period. The utilization of biphasic calcium phosphate in scaffold production is advantageous, as the rapid dissolution of TCP creates more space for new bone formation. At the same time, HA maintains the proper microarchitecture during the repair process^8^.

In a case series reported by Mekcha, Wongpairojpanich^2^, the utilization of a 3D bone block for horizontal bone defects was documented. This involved employing a combination of a 3D powder printing process and a low-temperature phase transformation to generate a unique low-crystalline nano-HA structure. The authors reported a high complication rate in the early 2 months as soft tissue perforation for the HA block graft alone (two out of three cases). To address this issue, the authors utilized CGFs and PRF membranes to promote soft tissue healing, resulting in no complications in the other cases. After 6 months, cone beam computed tomography (CBCT) images showed sufficient bone volume for implant placement in all patients. The mean maximum horizontal bone gain reported (3.56 ± 0.45 mm) was comparable to that achieved with autologous bone block grafts combined with particulate xenografts covered with a resorbable membrane (4.8 ± 0.79 mm)^27^ and allogeneic blocks (2.6 ± 2.5 mm)^28^.

Another material used in the fabrication of 3D-printed scaffolds for bone regeneration is calcium phosphate cement (CPC). CPCs are hydraulic cement comprising one or more calcium orthophosphate powders and a liquid phase^18^. This material is biologically active and osteoconductive, facilitating the migration and proliferation of osteoblasts, while its slow biodegradability ensures high volume stability over many years^18^. Moreover, calcium phosphate cements can be a carrier for specific agents to promote bone regeneration. Another advantage of CPC lies in its moldability and printability, allowing for base material modifications^18^. Schulz, Holtzhausen^18^ evaluated a 3D-printed CPC scaffold for sinus grafting. Nine months post-implantation, evident scaffold integration was observed, and implant placement was feasible with sufficient primary stability. According to the authors, one notable advantage of utilizing 3D-printed scaffolds is the ability to customize pore size according to biological and mechanical demands, which vary between non-load-bearing regions such as the maxillary sinus and load-bearing areas in the mandible. A recent histomorphometric analysis of human biopsies revealed that increased packing density reduced integration of bone substitute particles in sinus floor augmentation^29^. This issue could be addressed using a 3D-printed scaffold, as pore size is adjustable to optimize bone ingrowth^18^.

It has been suggested that the scaffold's ideal degradation rate aligns with the osteogenesis rate. Consequently, the scaffold's framework would be substituted by newly formed bone^18^. However, the studies reviewed found that the scaffolds were not entirely replaced by new bone within a short follow-up period (ranging from 1 month to 2 years). For instance, a complete replacement was not found even in the case 7 years and 3 months of the longest follow-up, although new bone formation was partly seen inside of the scaffolds^8^. Nonetheless, the 3D-printed scaffolds reported in this review provided durable support and achieved adequate bone union between the artificial bones and host bone tissues. This outcome may be attributed to the ability of 3D-printed scaffolds to be customized to fit the defect site, facilitating close bone-scaffold contact. Unlike conventional artificial bones, which typically require considerable surgical time and expertise for dimensional adjustment and fixation^6^, the 3D-printed scaffolds necessitated minimal or no dimensional adjustment (several minutes) and fixation, as reported by all included studies.

Porosity and pore size play crucial roles in cell attachment, differentiation, and proliferation. While increased material porosity can enhance cell adhesion and proliferation through interconnected pores, higher pore size or porosity may compromise mechanical resistance^18^. Ideally, scaffolds should possess an interconnected macroporous structure (>100 mm in diameter) to facilitate cell infiltration, bone growth, and neovascularization^30^. Incorporating microporosities into the macroporous structure can enhance permeability and cell migration, facilitating faster bone ingrowth and reducing patient healing time^18^. Notably, 3D manufacturing technology allows for scaffold customization to address the needs of each bone defect^30^. Conversely, allogeneic and xenogeneic materials have predetermined properties and lack adjustability^18^. In this review, pore sizes ranged from 300 to 2250 μm, with the porosity of 3D-printed scaffolds varying from 50 to 80%.

According to Saijo, Igawa^6^, inkjet printing technology stands out compared to other layer manufacturing methods due to its capability to process biocompatible and biodegradable materials at room temperature. This capability enables the creation of porous scaffolds with precisely controlled internal structures and excellent resolution. In contrast, the current stereolithography approach relies on photosensitive resin and photoinitiators, which typically lack biocompatibility or biodegradability. Additionally, this method generates radicals that can pose toxicity risks to human tissues^6^. Similarly, although less precise, fused deposition modeling utilizes thermoplastics that are often neither biocompatible nor biodegradable and may require support structures in specific scenarios^6^.

Some postoperative complications were described in the studies, including wound dehiscence, graft exposure, and local infection. The 3D-printed scaffold should be covered with a durable and thick flap to prevent these complications. A meticulous debridement of remaining unhealthy tissue inside the defect should also be carried out to avoid wound complications^12^. Moreover, Kanno, Nakatsuka^23^ it noted that the excessive height of the 3D-printed scaffold in contact with the recipient bone led to unexpected mobility and instability of the graft, increasing susceptibility to infection associated with friable granulation tissue. According to these authors, fixation of the 3D-printed scaffold to the recipient's bones helped reduce the incidence of postoperative infection.

Although the included studies reported promising results with the use of 3D-printed scaffolds in maxillofacial defect reconstruction, caution should be exercised in their evaluation. Limitations of this scoping review include the predominance of case series and case reports with short follow-up periods among the included studies. Additionally, only one RCT has been identified in the literature thus far. No comparisons between the efficacy of 3D-printed scaffolds and autologous bone (considered the gold standard) in bone formation have been reported thus far. Furthermore, bone formation was primarily assessed solely by CT images in most of the included studies.

Therefore, future studies should comprise larger sample sizes and longer follow-up periods to validate the efficacy of 3D-printed scaffolds in bone regeneration. It is also necessary to investigate variations in scaffold porosity and conduct long-term examinations of scaffold resorption characteristics to optimize stability and bone ingrowth. RCTs comparing 3D-printed scaffolds with autologous bone in bone regeneration should be conducted to confirm their efficacy.

In conclusion, custom-made 3D-printed bioresorbable scaffolds, seem to promote bone regeneration, offering a promising alternative for maxillofacial reconstruction due to their accuracy, osteoconductivity, and biocompatible properties.

## Figures and Tables

**Appendix:**
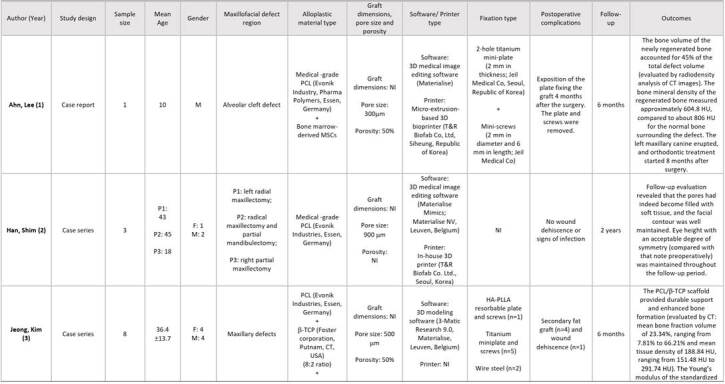
Characteristics of the studies and participants included in the review.

NI: not informed; MSCs: mesenchymal stem cells; PCL: polycaprolactone; HU: hounsfield unit; (-TCP: beta tricalcium phosphate; (-TCP: alfa tricalcium phosphate; HA-PLLA: Hydroxyapatite/poly-l-lactide; DBM: Demineralized bone matrix; CGFs: concentrated growth factors; PRF: platelet-rich fibrin; ISQ: implant stability quotient; nHA: nanohydroxyapatite; HA: hydroxyapatite; TCP: tricalcium phosphate; rhPDGF-BB: recombinant human platelet-derived growth factor BB; PRP: platelet-rich plasma; rhBMP-2: recombinant human bone morphogenetic protein-2.

1. Ahn G, Lee JS, Yun WS, Shim JH, Lee UL. Cleft Alveolus Reconstruction Using a Three-Dimensional Printed Bioresorbable Scaffold With Human Bone Marrow Cells. The Journal of craniofacial surgery. 2018;29(7):1880-3.

2. Han HH, Shim JH, Lee H, Kim BY, Lee JS, Jung JW, et al. Reconstruction of Complex Maxillary Defects Using Patient-specific 3D-printed Biodegradable Scaffolds. Plast Reconstr Surg Glob Open. 2018;6(11):e1975.

3. Jeong WS, Kim YC, Min JC, Park HJ, Lee EJ, Shim JH, Choi JW. Clinical Application of 3D-Printed Patient-Specific Polycaprolactone/Beta Tricalcium Phosphate Scaffold for Complex Zygomatico-Maxillary Defects. Polymers. 2022;14(4).

4. Kanno Y, Nakatsuka T, Saijo H, Fujihara Y, Atsuhiko H, Chung UI, et al. Computed tomographic evaluation of novel custom-made artificial bones, "CT-bone", applied for maxillofacial reconstruction. Regenerative therapy. 2016;5:1-8.

5. Mekcha P, Wongpairojpanich J, Thammarakcharoen F, Suwanprateeb J, Buranawat B. Customized 3D printed nanohydroxyapatite bone block grafts for implant sites: A case series. Journal of prosthodontic research. 2023;67(2):311-20.

6. Mangano C, Giuliani A, De Tullio I, Raspanti M, Piattelli A, Iezzi G. Case Report: Histological and Histomorphometrical Results of a 3-D Printed Biphasic Calcium Phosphate Ceramic 7 Years After Insertion in a Human Maxillary Alveolar Ridge. Frontiers in bioengineering and biotechnology. 2021;9:614325.

7. Rasperini G, Pilipchuk SP, Flanagan CL, Park CH, Pagni G, Hollister SJ, Giannobile WV. 3D-printed Bioresorbable Scaffold for Periodontal Repair. J Dent Res. 2015;94(9 Suppl):153s-7s.

8. Saijo H, Igawa K, Kanno Y, Mori Y, Kondo K, Shimizu K, et al. Maxillofacial reconstruction using custom-made artificial bones fabricated by inkjet printing technology. J Artif Organs. 2009;12(3):200-5.

9. Schuckert KH, Jopp S, Teoh SH. Mandibular defect reconstruction using three-dimensional polycaprolactone scaffold in combination with platelet-rich plasma and recombinant human bone morphogenetic protein-2: de novo synthesis of bone in a single case. Tissue Eng Part A. 2009;15(3):493-9.

10. Schulz MC, Holtzhausen S, Nies B, Heinemann S, Muallah D, Kroschwald L, et al. Three-Dimensional Plotted Calcium Phosphate Scaffolds for Bone Defect Augmentation-A New Method for Regeneration. Journal of Personalized Medicine. 2023;13(3).

**Appendix:**
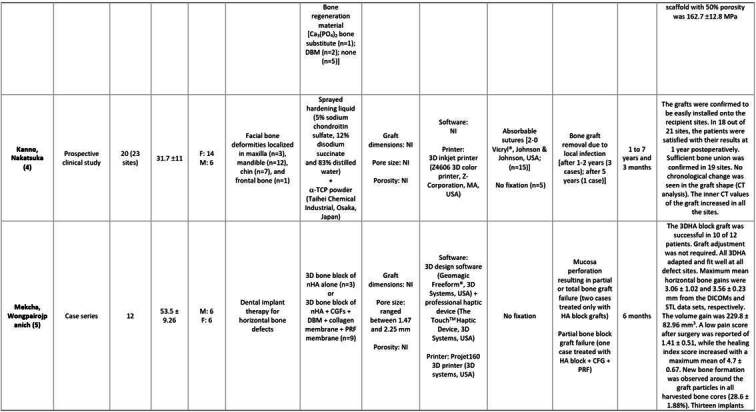
continuation

**Figure:**
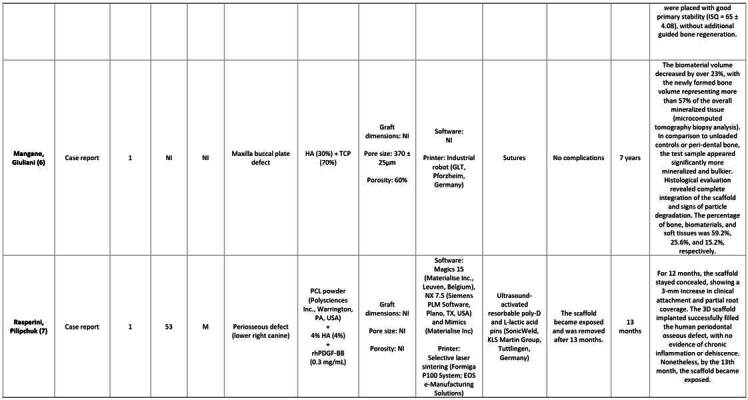
continuation

Appendix-

**Appendix-:**
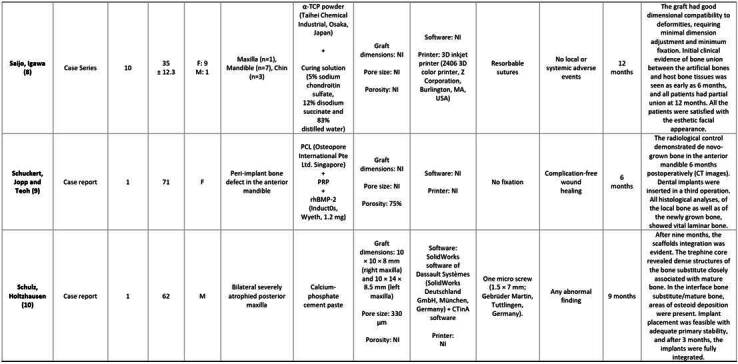
continuation
